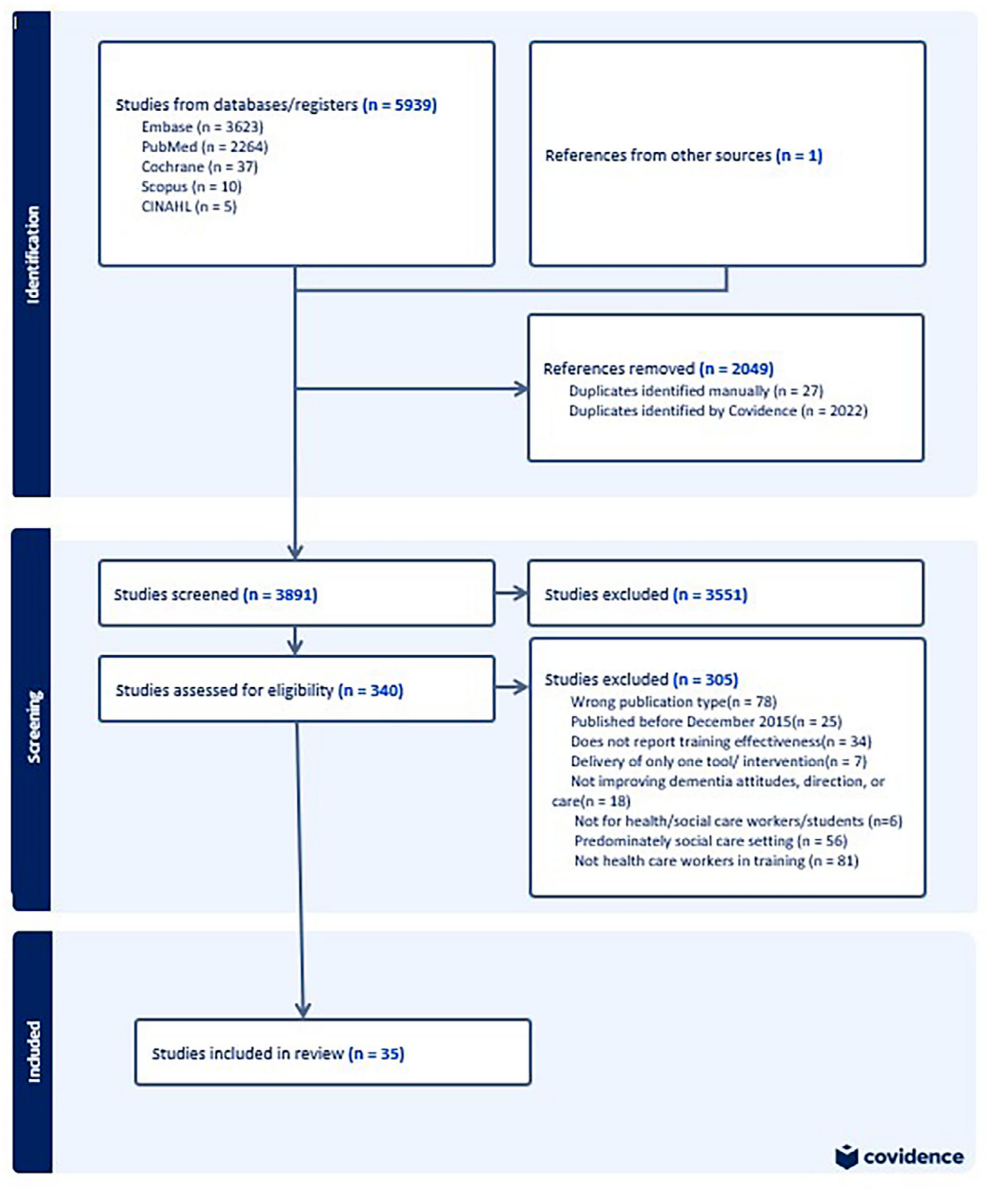# Dementia Education and Training for the Multidisciplinary Student Healthcare Workforce: A Systematic Review

**DOI:** 10.1002/alz70858_100783

**Published:** 2025-12-25

**Authors:** Malvika Muralidhar, Saskia Delray, Claudia Cooper, Sedigheh Zabihi, Sube Banerjee, Clarissa Giebel, Karen Harrison‐Dening, Yvonne Birks, Charlotte Kenten, Madeleine Walpert

**Affiliations:** ^1^ Queen Mary University of London, London, United Kingdom; ^2^ University of Nottingham, Nottingham, United Kingdom; ^3^ University of Liverpool, Liverpool, United Kingdom; ^4^ NIHR ARC NWC, Liverpool, Merseyside, United Kingdom; ^5^ Dementia UK, LONDON, United Kingdom; ^6^ University of York, York, United Kingdom; ^7^ Dementia UK, Cambridge, United Kingdom

## Abstract

**Background:**

Globally, dementia is a leading cause of mortality and morbidity, necessitating high‐quality care across healthcare specialties. Only a few education and training programmes developed to enhance healthcare students’ dementia care competencies are evidence based. We aimed to systematically review existing evidence on the effectiveness of dementia education and training for health and social care students.

**Methods:**

We searched five electronic databases for primary research studies (published between 2015‐2024), and unpublished grey literature, evaluating dementia training for health and social care students. We assessed risk of bias using the Mixed Methods Appraisal Tool (MMAT), prioritising studies scoring 4+ (higher quality) that reported significant findings on primary outcomes from controlled intervention trials. We reported outcomes using Kirkpatrick's framework. Professional stakeholders were consulted in focus groups regarding how findings might inform practice.

**Results:**

The included studies spanned 13 countries across 4 continents, covering diverse training programmes such as experiential learning, placement‐based, online learning, simulation, and classroom‐based approaches. 15/35 eligible studies were rated 4+ on the MMAT; only one met our a priori criteria for priority evidence. An experiential programme for UK medical students, “Time for Dementia”, which combined skill‐learning and reflective sessions with visits to people with dementia, was found to improve attitudes and knowledge over two years of participation, with qualitative studies supporting these findings. Asynchronous, self‐directed learning did not improve learning outcomes, relative to standard training. None of the programmes evaluated patient outcomes as an impact of students’ training. Nine focus group attendees agreed that the evidence reflected their experiences that consistent support, combined with skills‐based and reflective sessions, optimised student learning from initial patient‐focused encounters.

**Conclusions:**

Effective interventions increased confidence and enjoyment of dementia care encounters, and interest in dementia specialty careers. Mandating evidence‐based dementia skills programmes across specialties could ensure that students learn the skills and competencies required to be part of an effective future workforce and drive improvements in care quality. The findings offer valuable insights into developing a skilled dementia care workforce, with implications for both UK and international policy. Future research should incorporate patient perspectives to better evaluate the impact of educational programmes.